# The diagnostic accuracy of infrared thermography in lumbosacral radicular pain: a prospective study

**DOI:** 10.1186/s13018-024-04910-w

**Published:** 2024-07-17

**Authors:** Hong Liu, Zhaoji Zhu, Xiaohong Jin, Peng Huang

**Affiliations:** 1 Department of Anesthesiology and Pain, The First Affiliated Hospital of Soochow University, Pinghai Road NO. 899, Suzhou, Jiangsu China; 2 Department of General Practice, Changshu Hospital Affiliated to Soochow University, Suzhou, Jiangsu China

**Keywords:** Infrared thermography, Lumbosacral radicular pain, Diagnostic accuracy study

## Abstract

**Background:**

To identify the sensitivity, specificity, and overall diagnostic accuracy of infrared thermography in diagnosing lumbosacral radicular pain.

**Methods:**

Patients sequentially presenting with lower extremity pain were enrolled. A clinical certainty score ranging from 0 to 10 was used to assess the likelihood of lumbosacral radicular pain, with higher scores indicating higher likelihood. Infrared Thermography scans were performed and the temperature difference (ΔT) was calculated as ΔT = T1 - T2, where T2 represents the skin temperature of the most painful area on the affected limb and T1 represents the skin temperature of the same area on the unaffected limb. Upon discharge from the hospital, two independent doctors diagnosed lumbosacral radicular pain based on intraoperative findings, surgical effectiveness, and medical records.

**Results:**

A total of 162 patients were included in the study, with the adjudicated golden standard diagnosis revealing that 101 (62%) patients had lumbosacral radicular pain, while the lower extremity pain in 61 patients was attributed to other diseases. The optimal diagnostic value for ΔT was identified to fall between 0.8℃ and 2.2℃, with a corresponding diagnostic accuracy, sensitivity, and specificity of 80%, 89%, and 66% respectively. The diagnostic accuracy, sensitivity, and specificity for the clinical certainty score were reported as 69%, 62%, and 79% respectively. Combining the clinical certainty score with ΔT yielded a diagnostic accuracy, sensitivity, and specificity of 84%, 77%, and 88% respectively.

**Conclusion:**

Infrared thermography proves to be a highly sensitive tool for diagnosing lumbosacral radicular pain. It offers additional diagnostic value in cases where general clinical evaluation may not provide conclusive results.

**Trial registration:**

ChiCTR2300078786, 19/22/2023.

## Introduction

Lower extremity pain is a prevalent and debilitating issue that affects at least 20% of the adult population and 50% of seniors [1]. It stems from a variety of sources within the back and lower limb regions, including muscles, bones, and blood vessels, leading to a wide range of differential diagnoses. A common culprit behind this type of pain is lumbosacral radicular pain, frequently associated with nerve root compression due to lumbar disc herniation or degenerative conditions like foramen stenosis or lateral recess stenosis. Characteristically, this pain extends down the lower limb, following the path of one or more lumbar or sacral dermatomes [2, 3]. It may also present with signs of radicular irritation or diminished function [4].

In clinical practice, diagnosing lumbosacral radicular pain primarily relies on the identification of lower limb radicular symptoms, complemented by physical examinations such as a positive straight leg raise test, sensory and motor assessments, and imaging examinations like magnetic resonance imaging (MRI) and computer tomography (CT) to verify the nerve root compression [5, 6]. However, the research on the diagnostic precision of clinical neurological tests is limited, and the effectiveness of many physical exams for indicating lumbosacral radicular pain is generally poor [7, 8]. Furthermore, the utility of patient history and physical assessments for improved diagnosis is compromised by the absence of a widely accepted standard for comparison [9].

Medical imaging, specifically MRI, is a preferred technique for detecting herniated discs or lumbar spinal stenosis due to its superior soft tissue visualization capabilities and minimal radiation exposure [5]. Despite this, the sensitivity and specificity of MRI and CT in diagnosing herniated discs are relatively modest [10]. Studies have shown that herniated discs in around 20–76% of asymptomatic individuals [11, 12]. Additionally, there is minimal correlation between the intensity of radiculopathy (radicular pain), the size of the herniated disc, and the severity of lumbar spinal stenosis [13].

In the realm of healthcare diagnostics, infrared thermography (IT) has emerged as an increasingly favored assessment modality. By evaluating the variations in temperature distributions across matched limbs and trunks, IT has demonstrated efficacy in identifying potential health issues. Its application has been particularly effective in the diagnosis of rheumatic diseases, complex regional pain syndrome, and vascular disease [14–16]. Regarding lumbosacral radicular pain, evidence indicates that IT can detect a significant reduction in skin temperature decreases on the affected side compared to the contralateral, non-affected side, particularly in instances of lumbar and cervical disc herniation [17, 18]. The utilization of infrared thermography (IT) for diagnosing lumbosacral radicular pain relies on the concept that compression or irritation of nerve roots can impact sympathetic nerve activity, subsequently affecting vasomotor control and resulting in alterations in skin temperature [19]. Nerve root compression can disrupt the usual sympathetic regulation of blood flow, potentially causing localized hypothermia or hyperthermia. These temperature variations can be identified through IT and utilized as a non-invasive diagnostic marker for nerve root pathology. Despite the promising utility of IT in this context, diagnostic accuracy studies have reported various sensitivity (from 0.84 to 1.0) and specificity (from 0.0 to 1.0), underscoring the variability in outcomes [20]. Notably, these investigations have been criticized for methodological shortcomings, including biased interpretation tests, inappropriate cohort selection, insufficient clinical descriptions, and small sample sizes [21–25].

To evaluate the diagnostic value of IT for radiculopathy, a comprehensively designed diagnostic accuracy study was conducted. The study aimed to identify the optimal temperature differential threshold between the affected and unaffected sides that would accurately reflect the sensitivity, specificity, and overall diagnostic accuracy of IT in diagnosing lumbosacral radicular pain.

## Methods

### Study design and participants

In this prospective cohort study, we enrolled patients sequentially presenting with lower extremity pain, with or without concomitant back pain as the primary symptom. Exclusion criteria were bilateral symptoms, a history of spine or lower limb surgery, or any contraindications to undergoing MRI or CT examinations. The study adhered to the ethical standards of the Declaration of Helsinki and received approval from the ethics committee of our institution(2023 − 504). All participants provided written informed consent prior to their inclusion in the study. Furthermore, this trial was duly registered with the Chinese Clinical Trial Registry (ChiCTR2300078786, 19/22/2023).

The manuscript preparation process was guided by the STARD [26].

### Clinical evaluation of patients

Enrolled patients underwent an initial thorough evaluation, including reviewing their medical history, physical examination, and imaging tests (3D-CT scan, MRI, X-ray.) for the lumbar spine. A pain specialist provided a clinical certainty score ranging from 0 to 10 to assess the likelihood of lumbosacral radicular pain as the source of lower extremity pain based on the medical history, physical findings, and imaging results. Following the initial assessment, further diagnostic tests such as blood tests, lower extremity electromyography, vascular ultrasound, and selective nerve root block, were recommended to refine the diagnosis.

### Reference (‘Gold’) standard for radiculopathy diagnosis

The ‘gold’ standard for diagnosing radiculopathy involved two independent surgeons specializing in endoscopic lumbar spine surgery. These surgeons performed endoscopic lumbar decompression on consenting patients, targeting one or two spine segments based on the specific pain radiation area and the findings from MRI and physical examinations. The presence of lumbar and lumbosacral radicular pain was corroborated by intraoperative evidence of nerve root compression, manifested through nerve root displacement, ischemia, restricted mobility due to the impingement by disc herniation or ligamentum flavum hypertrophy and lateral recess stenosis. Successful decompression allowed for the restoration of nerve root function and position.

Upon the patient’s discharge from the hospital, the same specialists thoroughly reviewed the patient’s medical records, which included medical history, pain symptoms, the effectiveness of decompression, follow-up (up to a month after surgery if needed), hematology examination (blood routine, blood biochemistry, blood coagulation test, erythrocyte sedimentation rate, and C-reactive protein), physical examinations (straight leg elevation test, sensory test, muscle strength test, Achilles tendon reflex, knee reflex, and pathological signs), image examinations (lumbar spine 3D-CT scan, lumbar spine MRI, and lumbar spine X-ray), as well as the results of lower extremities electromyography and selective nerve root block. The diagnosis was finalized by integrating surgical findings with the comprehensive medical documentation.

### IT scan and temperature measurement

All patients received an IT scan administered by an independent physician using IRIS-5000 (Medicore, Seoul, Korea). This device captured infrared radiation emitted from the body surface, visualizing thermal variances across 16 color levels and pixel density. The ambient temperature in the IT room was maintained at 25 °C. Prior to the examination, patients were required to acclimate for 15 min in the room, devoid clothing on their back and lower extremities, with strict instructions to avoid any skin contact or manipulation during this period. The IT scanning process encompassed five perspectives: posterior and anterior of the back and lower extremities, right and left lateral views of the lower extremities, and a plantar view. The results were analyzed by comparing temperatures in the regions of most pronounced pain against the equivalent zones on the opposite limb. The temperature difference (ΔT) was calculated using the formula ΔT = T1 – T2, where T1 represents the skin temperature on the same area of the unaffected limb, and T2 represents the temperature in the area of greatest pain on the affected limb. Consequently, a positive difference indicates a lower temperature in the symptomatic area, suggesting potential pathology.

### Primary outcome

The primary outcome of this study focuses on evaluating the sensitivity, specificity, and diagnostic accuracy of the temperature difference (ΔT) between the affected and unaffected sides in diagnosing lumbosacral radicular pain. As a secondary objective, the study seeks to determine the impact of integrating the temperature difference data with a clinical certainty score on enhancing the diagnostic precision for lumbosacral radicular pain, based on the medical history, physical examination, and imaging studies, would enhance diagnostic accuracy.

### Statistical analysis

The determination of our study’s sample size was informed by prior research outcomes indicating a sensitivity of 0.8 and a specificity of 0.7 for IT in diagnosing lumbosacral radicular pain [20]. Additionally, the prevalence of lumbosacral radicular pain among individuals with lower extremity pain was reported to be 0.6 [27]. Utilizing these parameters, our calculations established that a minimum cohort of 162 patients was required to achieve the desired stability and validity for our investigation.

Statistical analysis was conducted using the RStudio 2023 software package. For data representation, categorical variables were expressed as counts and percentages, whereas continuous variables were presented as either medians and interquartile ranges or means and standard deviations, depending on their distribution patterns. Initially, Generalized Additives Models were applied to assess the linear or non-linear relationship between the temperature difference (ΔT) and the likelihood of lumbosacral radicular pain. In the case where a non-linear relationship was observed, a threshold effect analysis was conducted using a two-piecewise linear regression model to better delineate this relationship. Further, segmented regression, also known as piece-wise regression, was utilized to model the data with distinct linear segments for each interval. The presence of a threshold effect was evaluated through a log-likelihood ratio test, comparing the one-line (non-segmented) model against the segmented regression model. The inflection point, which signifies the transition between segments, was identified using the model that yielded the highest likelihood, determined by a two-step recursive method. The diagnostic accuracy of ΔT within the determined inflection points was quantified following the log-likelihood ratio test outcomes. The optimal threshold for ΔT, aimed at maximizing both sensitivity and specificity for diagnosing lumbosacral radicular pain, was pinpointed on the receiver operating characteristic (ROC) curve. Subsequently, the diagnostic accuracy of ΔT was compared to the clinical certainty score using the McNemar test. A logistic regression was developed to integrate the clinical certainty score with ΔT for a refined prediction of the diagnosis. The area under curve (AUC) comparisons for statistical discrepancies were made using Delong’s method. Lastly, Pearson and point-biserial correlation analyses were applied to explore the relationships between ΔT, pain intensity, neurologic signs, and MRI.

## Results

### Study population

Between October 2023 and February 2024, our study screened a cohort of 174 individuals presenting with symptoms indicative of lumbosacral radicular pain. Of these, 4 patients were excluded due to being unable to undergo MRI because of a heart stent, and 5 patients were excluded owing to previous lumbar spine fusion surgery. Additionally, 3 patients exhibited bilateral symptoms, leading to their exclusion from the study. Ultimately, 162 patients were enrolled in the study. Within the patient group, 17 experienced pain in the buttocks, while 100 patients reported pain radiating below the knee. Additionally, 117 patients reported lower back pain. The demographic and clinical baseline characteristics of these patients are detailed in Table 1.

### Final (golden standard) diagnosis

**Fig. 1:**
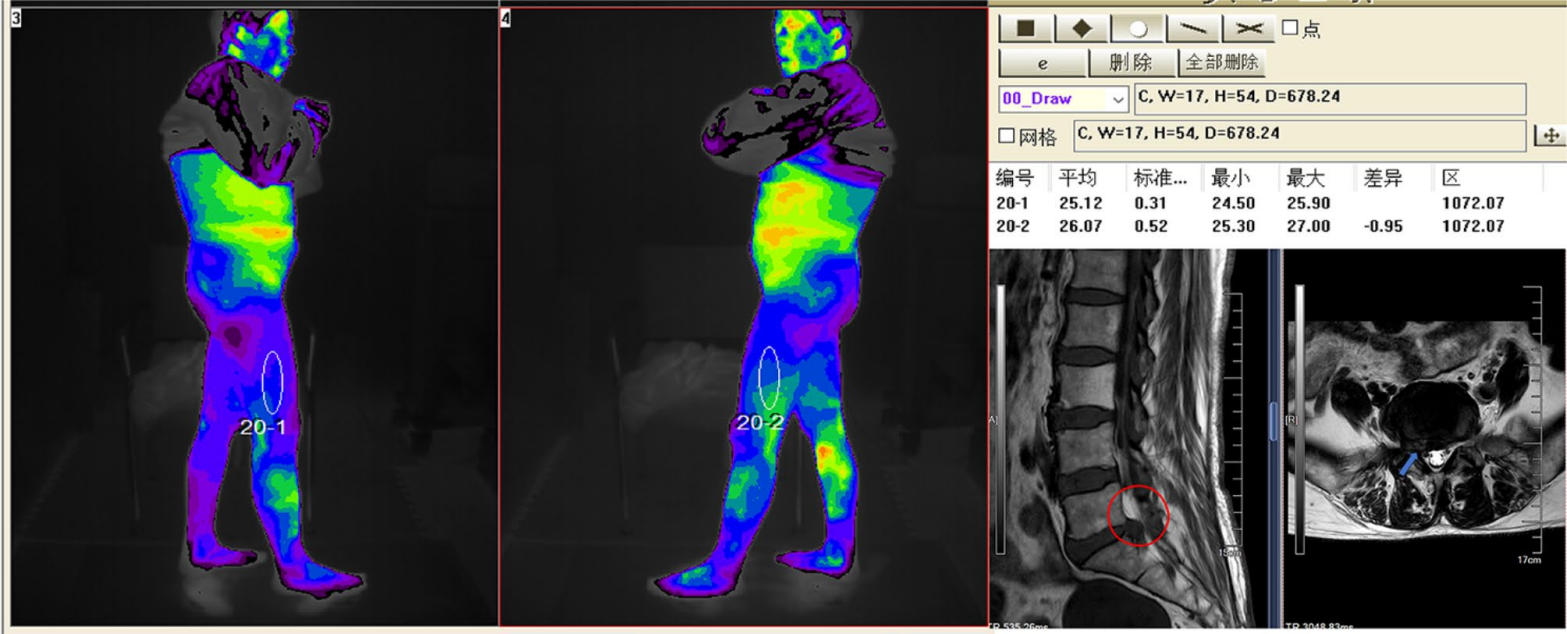
A 66-year-old female patient experienced radiating pain in her right lower limb for 20 days, predominantly in the outer thigh area. The IT examination showed a lower surface temperature in the affected area with a temperature difference of 0.95℃, compared to the unaffected side. A subsequent lumbar MRI revealed a lumbar disk herniation at the L5/S1 level on the right side, leading to nerve root compression. A percutaneous lumbar endoscopic discectomy was performed to address the issue, and during the surgery, the S1 nerve root was found to be closely associated with the intervertebral disc, exhibiting signs of ischemia and poor mobility. The successful decompression resulted in significant alleviation of the patient’s lower limb pain

A total of 122 (75%) patients underwent surgery, with 21 patients being diagnosed with a different disease. The remaining 101 (62%) patients were ultimately diagnosed with lumbosacral radicular pain caused by lumbar disc herniation based on the surgical results. For all the 61 patients without lumbosacral radicular pain, alternate diagnoses were performed, and piriformis syndrome was identified in10 cases, herpes zoster neuralgia was found in 4 cases, lumbar facet joint disorder was in 8 cases, femoral head necrosis was in 2 cases, myofasciitis was in 10 cases, gluteal epithelial neuritis was in 2 cases, plantar fasciitis was in 3 cases, peroneal neuritis was in 3 cases, ankle tunnel syndrome was in 2 cases, lung cancer bone metastasis was in 1 case, diabetes neuralgia was in 5 cases, thromboangiitis obliterans was in 3 cases, knee and hip arthritis was in 2 cases, Sacroiliac arthritis was in 2 cases, osteoporosis was in 2 cases, and ankylosing spondylitis was in 2 cases Fig. 1.

### Relationship between ΔT and lumbosacral radicular pain

**Fig. 2:**
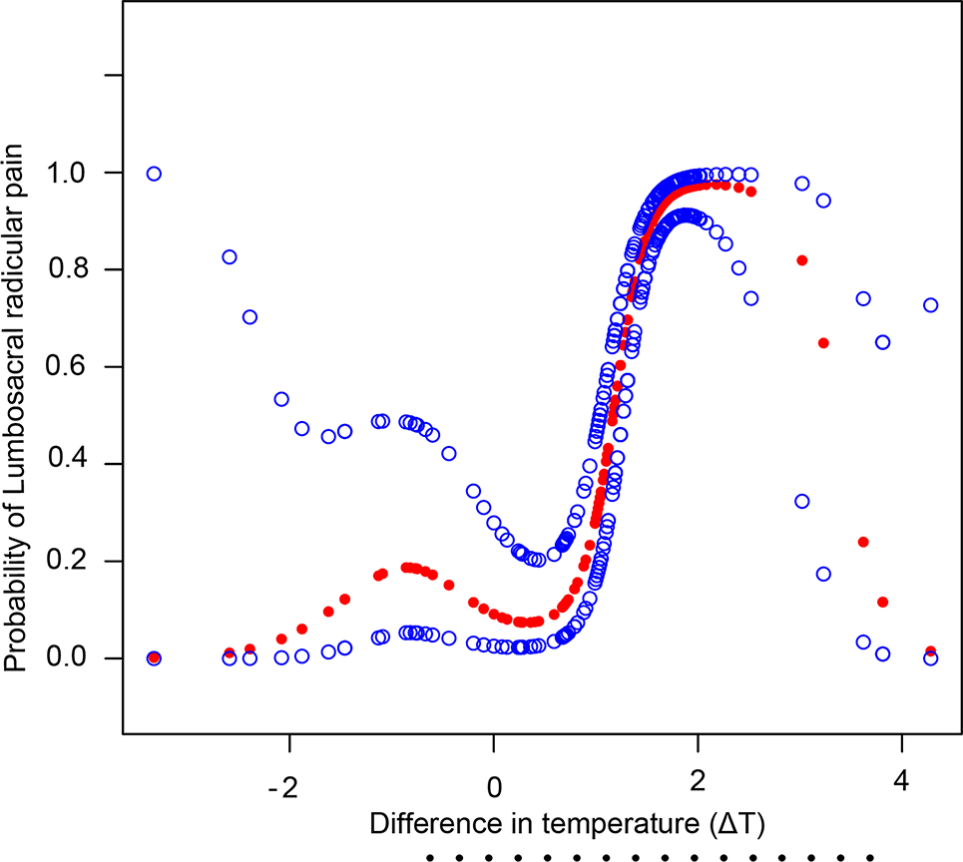
Generalized Additives Models demonstrate an S-shaped relationship between ΔT and the risk of lumbosacral radicular pain. As the temperature increased, the risk of lumbosacral radicular pain also increased within the range of ΔT from 0.8℃ to 2.2℃

In the cohort of patients diagnosed with radiculopathy, the temperature difference ΔT ranged from − 1.46℃ to 3.62. As shown in Fig. 2, the application of generalized additive models for smooth curve fitting illuminated an S-shaped curve illustrating the relationship between ΔT and the risk of developing lumbosacral radicular pain. Notably, within the ΔT range of 0.8 °C to 2.2 °C, there was a significant increase in the risk of lumbosacral radicular pain, with an odds ratio (OR) of 6.37 (95% Confidence Interval [CI]: 4.20–8.54, *P* < 0.001). This analysis underscores the predictive value of ΔT in assessing the likelihood of lumbosacral radicular pain, suggesting that specific temperature differentials may be indicative of an elevated risk for the condition.

### ΔT and clinical certainty score in the diagnosis of lumbosacral radicular pain

**Fig. 3:**
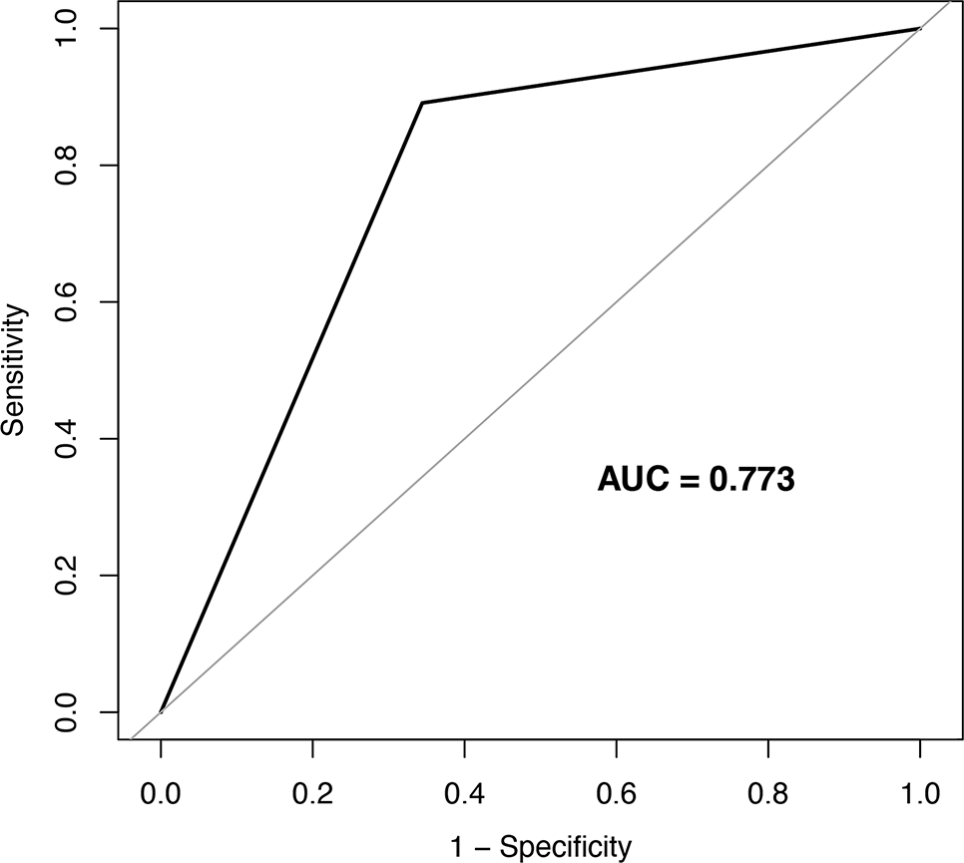
ROC curves for ΔT to diagnose lumbosacral radicular pain in patients with lower limb pain

Based on the generalized additive model, the optimal diagnostic range for ΔT was observed as 0.8℃ and 2.2℃. Within this range, the diagnostic accuracy, sensitivity, and specificity for ΔT for identifying lumbosacral radicular pain were computed to be 80%, 89%, and 66%, respectively. The ROC curve analysis yielded an AUC of 0.77 (95% CI 0.88 to 0.95) for ΔT in diagnosing the lumbosacral radicular pain (Fig. 3).

**Fig. 4:**
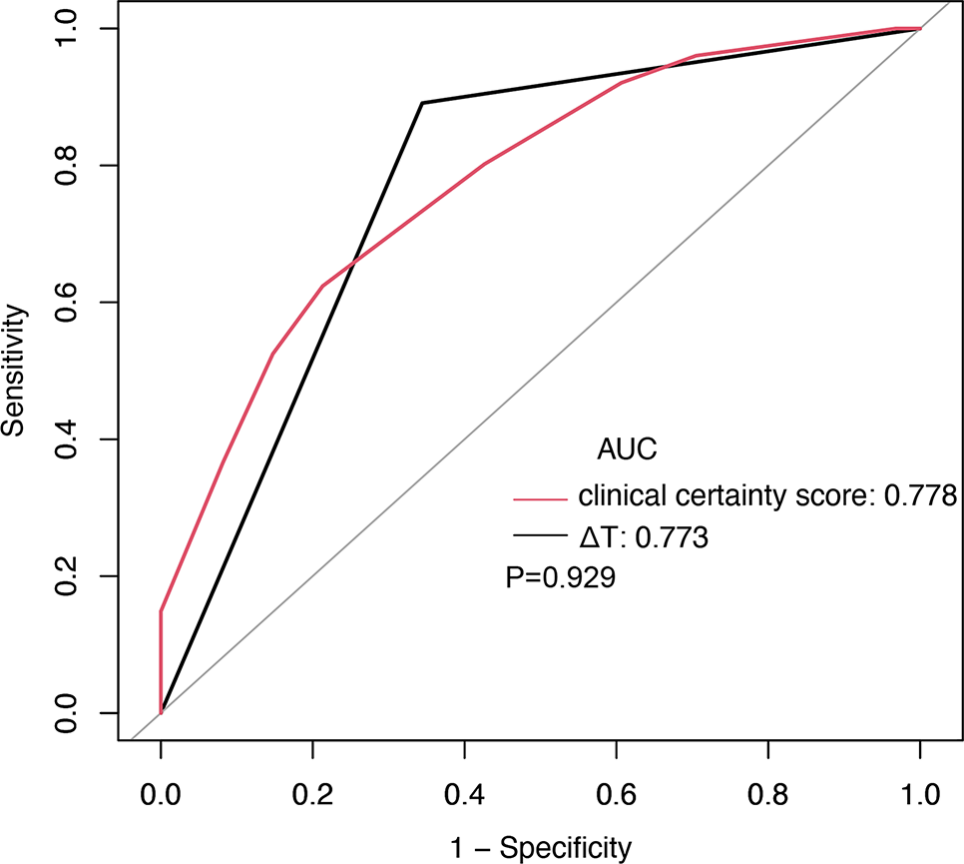
ROC curves for ΔT and clinical certainty score to diagnose lumbosacral radicular pain in patients with lower limb pain. There was no significant difference in AUC between the clinical certainty score and ΔT, *P* = 0.929

**Fig. 5:**
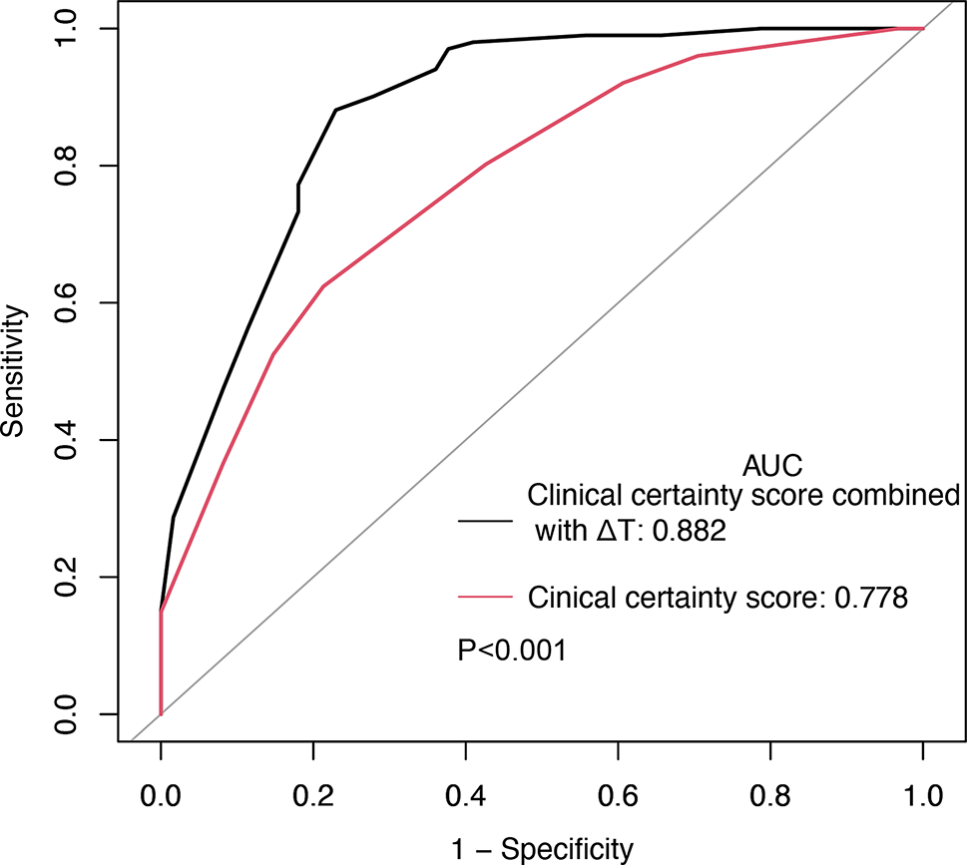
Comparison of AUC for clinical certainty score alone versus combined with ΔT, *P* < 0.001

In parallel, the AUC for the clinical certainty score was 0.78 (95% CI 0.71 to 0.85). The optimal cutoff of the clinical certainty score that achieved the highest diagnostic accuracy was 6, at which the diagnostic accuracy, sensitivity, and specificity were 69%, 62%, and 79%, respectively. Notably, although comparative analysis revealed no significant difference in AUC between the clinical certainty score and ΔT (0.78 vs. 0.77, *P* = 0.929) (Fig. 4), integrating ΔT with the clinical certainty score markedly enhanced diagnostic effectiveness, as evidenced by a combined AUC of 0.88, significantly surpassing the AUC for the clinical certainty score alone (0.88 vs. 0.78, *P* < 0.001) (Fig. 5; Table 2). This observation underscores the added value of incorporating physiological measurements with clinical evaluations in refining the diagnostic approach for lumbosacral radicular pain.

**Table 1:** Patients? demographics

Characteristics	Value
**No. of patients (n)**	162
**Age in years** (mean [range])	57.7 [23–89]
**Female sex** (n [%])	103 [64]
**Duration of disease in months** **(**mean [range]**)**	12.9 [0.5–240]
**Laterality** (n [%])	
Left	74 [46]
Right	88 [54]
**Pain location** (n [%])	
With back pain	117 [72]
Pain radiating above the knee	62 [38]
Pain radiating below the knee	100 [62]
**Physical examinations** (n [%])	
Positive straight leg raising	59 [36]
Asymmetric decrease in sensory response	50 [31]
Asymmetric decrease in motor response	38 [23]
Asymmetric decrease in reflexes	33 [20]
**Visual-analogue scale for pain (**mean**)**	**5.7**
**Nerve root compression on MRI** (n [%])	
Definite	32 [20]
Probable	55 [34]
Possible	46 [28]
Definitely not	29 [18]

**Table 2:** Diagnostic information for the clinical certainty score and ΔT

	Optimal cut-off value	Sensitivity	Specificity	Accuracy	PPV	NPV
Clinical certainty score	≥ 6	0.62	0.79	0.69	0.83	0.56
ΔT	0.8–2.2℃	0.89	0.66	0.80	0.81	0.78
Combined		0.88	0.77	0.84	0.86	0.80

Clinical certainty score: a scale from 0 to 10 used to evaluate the probability of lumbosacral radicular pain as the root cause of lower extremity pain. A higher score indicates a higher likelihood of lumbosacral radicular pain. This score is determined by an experienced pain doctor through a thorough assessment of the patient’s medical history, physical examination, and imaging studies

ΔT was calculated using the formula ΔT = T1 - T2, where T1 is the skin temperature of the same area on the unaffected limb and T2 is the skin temperature of the most painful area on the affected limb

PPV: Positive predictive value

NPV: Negative predictive value

### Diagnostic accuracy of ΔT between different duration of symptom

To assess the diagnostic accuracy of ΔT between different durations of symptoms, patients with lower limb radiating pain were categorized into acute (symptom duration less than 1 month), subacute (symptom duration between 1 and 3 months), and chronic (symptom duration more than 3 months) groups for subgroup analysis. The diagnostic accuracy, sensitivity, and specificity were found to be more optimal in subacute and chronic patients (Table 3).

**Table 3:** Diagnostic accuracy of ΔT between different duration of symptom

Duration of symptom	Sensitivity	Specificity	Accuracy	PPV	NPV	*P*-value
< 1month	0.50	1	0.6	1	0.33	0.414
1-3mon	0.84	0.72	0.80	0.85	0.72	< 0.001
> 3	0.69	0.7	0.70	0.91	0.35	< 0.001

### Relationship between ΔT and pain or neurologic signs

**Fig. 6:**
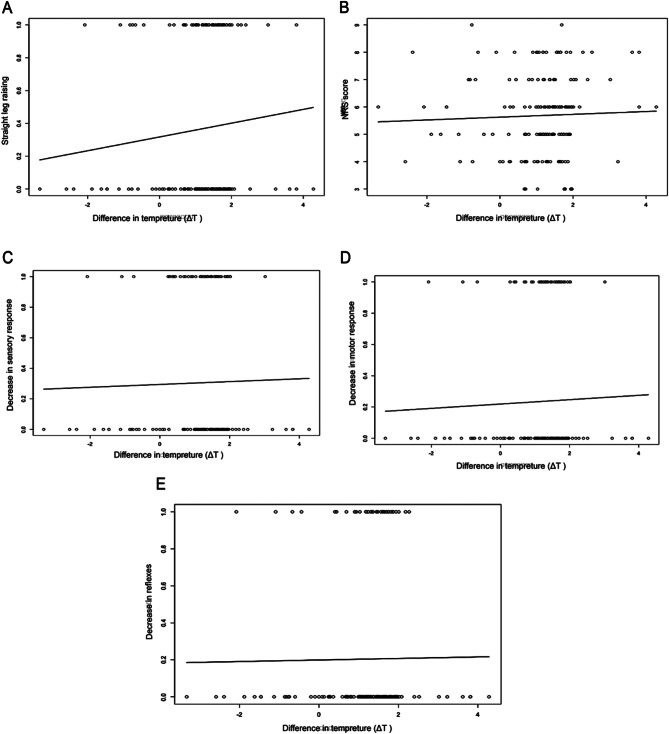
Relationships between ΔT and pain and neurologic signs. (**A**) The relationship between the temperature and the positive raising of the straight leg, *r* = 0.096, *P* = 0.217. (**B**) The relationship between the temperature and the pain intensity measured by NRS, *r* = 0.039, *P* = 0.619. (**C**) The relationship between the temperature and the decrease in sensory response, *r* = 0.023, *P* = 0.284. (**D**) The relationship between the temperature and the decrease in motor response, *r* = 0.037, *P* = 0.643. (**E**) The relationship between the temperature and the decrease in reflex, *r* = 0.012, *P* = 0.883

**Fig. 7:**
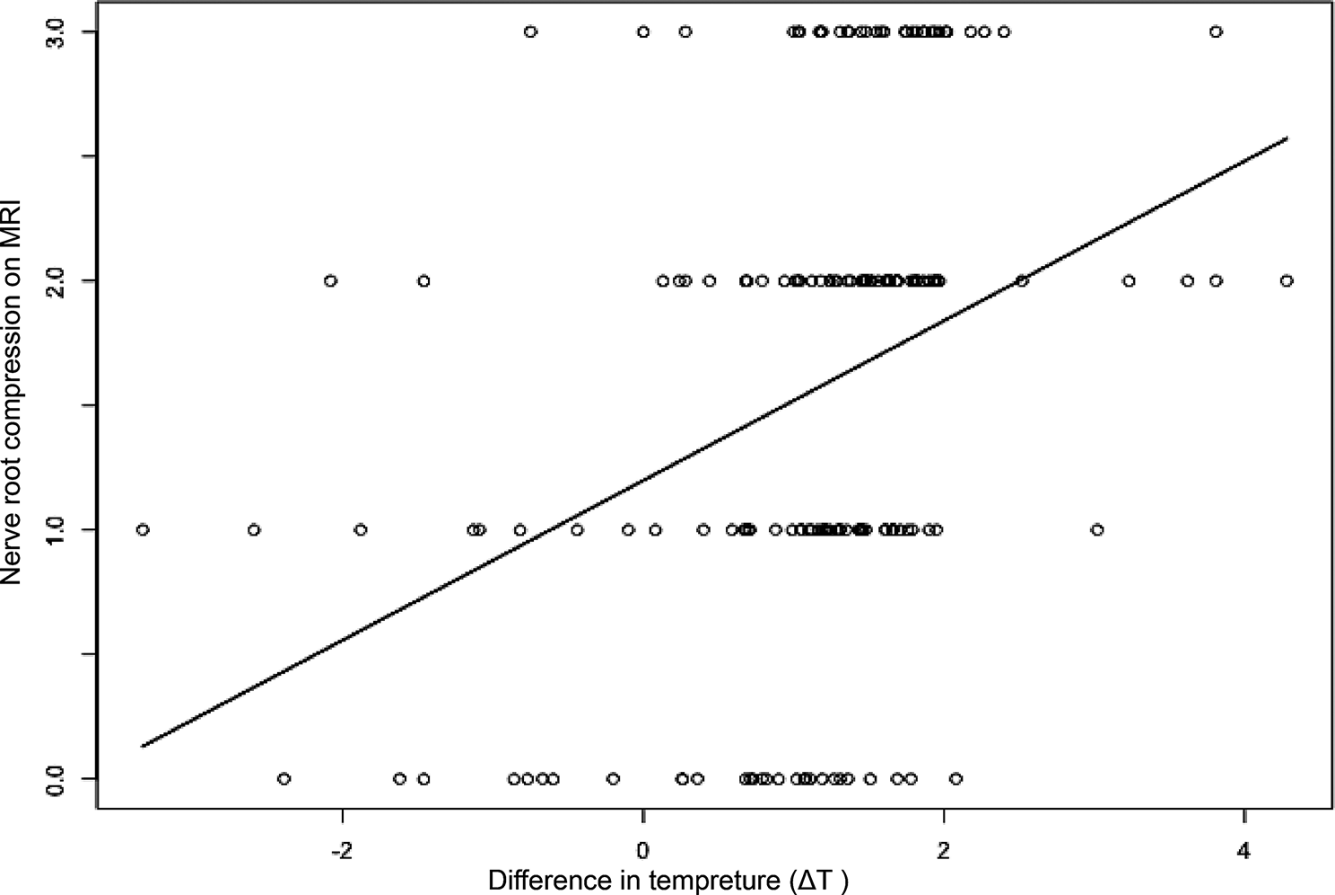
Positive linear correlation between temperature and the severity of nerve root compression on MRI, *r* = 0.359, *P* = 0.000

We further examined the relationship between ΔT and pain or neurologic signs. Contrary to expectations, we found no significant correlation between increased ΔT and higher Numerical rating scale (NRS) scores or neurologic deficits, such as s positive SLR, reduced sensory, and impaired motor responses (Fig. 6). However, a notable association emerged between ΔT and the severity of nerve root compression as determined by MRI. Specifically, as the severity of compression elevated, the temperature difference in the lower limbs of patients increased correlatively (Fig. 7).

## Discussion

Our research demonstrates that the measurement of temperature difference (ΔT) ranging from 0.8℃ to 2.2℃ using infrared thermography, offers a viable diagnostic tool for identifying lumbosacral radicular pain. The diagnostic performance of ΔT within this range is robust, with an accuracy of 80%, sensitivity of 89%, and specificity of 66%. The diagnostic efficacy of ΔT, as indicated by the AUC, aligns closely with that of the clinical certainty score. This score encapsulates a comprehensive clinical assessment, thus affirming the value of ΔT as a significant, standalone diagnostic indicator. In addition, our findings revealed enhanced diagnostic potential when combining ΔT with the clinical certainty score. This synergistic approach markedly improves the diagnostic AUC, suggesting that integrating quantitative thermal imaging with qualitative clinical evaluations can elevate the precision of diagnosing lumbosacral radicular pain.

The utility of thermography in diagnosing lumbosacral radicular pain has promoted considerable debate, evidenced by the variability in diagnostic accuracy reported across different studies. This discrepancy in the sensitivity and specificity of IT can be attributed to several factors, including divergences in the gold standard for diagnosis, the thermographic devices used, and the criteria established for identifying abnormalities. For instance, a study that evaluated 112 patients (56 with sciatica and 56 without), found that the sensitivity of thermography was 60% and the specificity was 48% [21]. Another study focused on the role of thermography in investigating nerve root compression caused by spinal stenosis, and found a concordance rate of only 48% with the final diagnosis, significantly lower than the concordance rates of myelography, computerized tomography, and electromyography, which each stood at 71% [28]. Conversely, Chafetz et al. presented a more favorable view of thermography in diagnosing nerve root compression. They used CT as the reference standard and concluded that thermography is a highly sensitive method for diagnosis, achieving a specificity of 60% and a remarkable sensitivity of 100% [29]. Similarly, Pochaczevsky et al. validated the correlation between thermographic findings and clinical as well as surgical observations in patients with nerve root compression [30].

Our research aligns with certain aspects of previous investigations while diverging from others, showing both scientific value and distinct advantages. One of the primary strengths of our study lies in the inclusion of a diverse group of patients with unilateral lower limb pain, enhancing the generalizability of our findings across the spectrum of lower extremity pain cases marked by diagnostic uncertainty. Moreover, our study is the first large observational cohort study dedicated to examining patients with unilateral lower extremity pain. It uniquely integrates intraoperative observations with post-surgical outcomes to assess nerve compression, addressing a gap in the existing literature where the utility of alternative imaging modalities and electromyography in diagnosing lumbosacral nerve compression has been contested [5, 7, 11, 31]. Therefore, the methodology employed for establishing the gold standard diagnosis in our study arguably offers a more robust and comprehensive framework compared to those utilized in prior research.

Disk herniation and spinal stenosis are commonly recognized causes of lumbosacral radicular pain. However, the presence of radiculopathy does not invariably correspond with nerve root compression observable on MRI, and imaging anomalies frequently do not always correlate with symptomatic pain [32, 33]. A meta-analysis has been conducted to summarize the diagnostic accuracy of imaging examinations compared to surgery (reference test) for lumbar disc herniation detection. The analysis revealed MRI exhibited a sensitivity range of 72.3-87.7% and a specificity span of 61.9-87.5%, which is comparable to CT [34]. In clinical practice, an accurate diagnosis of lumbosacral radicular pain relies largely on combined diagnostic methods. In this study, we developed a clinical certainty score evaluated by an experienced pain doctor. This score encapsulates an exhaustive evaluation of the patient’s medical history, physical examination results, and imaging findings, mirroring the diagnostic approach that is commonly adopted in clinical settings [35]. The method results in moderate diagnostic accuracy in our study, with a sensitivity of 62% and specificity of 79%, suggesting a considerable number of false positives and negatives of the method suggests that the diagnostic method has.

Integrating IT with clinical judgment significantly enhances the diagnostic precision for lumbosacral radicular pain, elevating the accuracy from 69 to 85%. It also improved the sensitivity from 62 to 88%, reducing the misdiagnosed false negative cases from 38 to only 12 and increasing the correctly identified positive patients from 63 to 89. The incorporation of IT into the clinical diagnostic process promises a more expedited and accurate identification of lumbosacral radicular pain, potentially streamlining the pathway to treatment and improving patient outcomes by minimizing diagnostic delays and associated costs. Regarding the relationship between ΔT and pain intensity or neurologic signs, previous research exploring plantar thermography’s correlation with low back pain intensity revealed a significant elevation in ΔT within the plantar region among subjects with severe low back pain, alongside a notable correlation with pain intensity (correlation coefficients of 0.502, *P* = 0.000) [16]. Despite these insights, our investigation didn’t establish a link between ΔT variations and either NRS pain scores or neurologic signs, suggesting a limited predictive utility of ΔT in examining pain or neurologic impairments. To the best of our knowledge, only one previous research was focused on the correlations between pain, neurologic signs, and the value of ΔT for assessing symptomatic severity. Aligns with our findings, their work reported low diagnostic accuracies of ΔT for pain and neurologic signs. Additionally, a moderate correlation was found between low back pain, leg pain, gait, SLR, sensory disturbance, motor weakness, and the temperature difference for the entire lower limb surface [22].

Several limitations apply to this study. Firstly, the study excluded patients with bilateral lower limb pain. However, there is a need for further research to assess the diagnostic effectiveness of thermography equipment in a broader range of lower limb radiating pain patients, particularly those with bilateral symptoms. Secondly, the high prevalence of lumbosacral pain (62%) within our cohort may influence the calculation of diagnostic accuracy. Future studies should consider including a control group of healthy individuals to provide a more balanced comparison and to better understand the diagnostic efficacy of IT. Thirdly, Our analysis was also limited to the temperature of areas identified by patients as most painful. Future research should assess whether temperature variations across affected dermatomes correlate with pain intensity and neurological signs to refine the diagnostic utility of thermography. Fourth, Further research comparing IT to other diagnostic tests in the preoperative setting is also necessary.

## Conclusion

The findings of this extensive study on consecutive patients with lower limb pain suggest that infrared thermography is a highly sensitive tool for diagnosing lumbosacral radicular pain. The accuracy of infrared thermography is similar to that of traditional clinical evaluation methods, which include the patient’s medical history, physical examination, and imaging studies. In situations where clinical evaluation may be inconclusive, infrared thermography appears to provide additional diagnostic value especially in subacute and chronic patients and also warrants further exploration in future research.

## Data Availability

The datasets used and/or analyzed during the current study are available from the corresponding author on reasonable request.

## Abbreviations

AUC: Area under curve
CT: Computer tomography
IT: Infrared thermography
MRI: Magnetic resonance imaging

## References

[1] Lucas JW, Connor EM, Bose J. Back, Lower Limb, and Upper Limb Pain among U.S. adults, 2019. NCHS Data Brief, 2021(415): p. 1–8. 34473621

[2] Bogduk N (2009). On the definitions and physiology of back pain, referred pain, and radicular pain. Pain.

[3] Tarulli AW, Raynor EM (2007). Lumbosacral radiculopathy. Neurol Clin.

[4] Van Boxem K (2010). 11. Lumbosacral radicular pain. Pain Pract.

[5] Kreiner DS (2014). An evidence-based clinical guideline for the diagnosis and treatment of lumbar disc herniation with radiculopathy. Spine J.

[6] Jensen RK (2019). Diagnosis and treatment of sciatica. BMJ.

[7] van der Windt DA et al. Physical examination for lumbar radiculopathy due to disc herniation in patients with low-back pain. Cochrane Database Syst Rev, 2010(2): p. Cd007431.10.1002/14651858.CD007431.pub220166095

[8] Tawa N, Rhoda A, Diener I (2017). Accuracy of clinical neurological examination in diagnosing lumbo-sacral radiculopathy: a systematic literature review. BMC Musculoskelet Disord.

[9] Peene L, et al. 1. Lumbosacral radicular pain. Pain Pract; 2023. 10.1111/papr.13317 37985718

[10] Wassenaar M (2012). Magnetic resonance imaging for diagnosing lumbar spinal pathology in adult patients with low back pain or sciatica: a diagnostic systematic review. Eur Spine J.

[11] Brinjikji W (2015). MRI findings of Disc Degeneration are more prevalent in adults with low back Pain than in asymptomatic controls: a systematic review and Meta-analysis. AJNR Am J Neuroradiol.

[12] Jensen MC (1994). Magnetic resonance imaging of the lumbar spine in people without back pain. N Engl J Med.

[13] el Barzouhi A (2013). Magnetic resonance imaging in follow-up assessment of sciatica. N Engl J Med.

[14] Lahiri BB (2012). Medical applications of infrared thermography: a review. Infrared Phys Technol.

[15] Branco JHL (2022). Clinical applicability of infrared thermography in rheumatic diseases: a systematic review. J Therm Biol.

[16] Adam M (2017). Computer aided diagnosis of diabetic foot using infrared thermography: a review. Comput Biol Med.

[17] Ra JY (2013). Skin temperature changes in patients with unilateral lumbosacral radiculopathy. Ann Rehabil Med.

[18] Zhang HY, Kim YS, Cho YE (1999). Thermatomal changes in cervical disc herniations. Yonsei Med J.

[19] Kim YC (2003). Infrared thermographic imaging in the assessment of successful block on lumbar sympathetic ganglion. Yonsei Med J.

[20] Hoffman RM, Kent DL, Deyo RA (1991). Diagnostic accuracy and clinical utility of thermography for lumbar radiculopathy. A meta-analysis. Spine (Phila Pa 1976).

[21] McCulloch J (1993). Thermography as a diagnostic aid in sciatica. J Spinal Disord.

[22] Takahashi Y, Takahashi K, Moriya H. Thermal deficit in lumbar radiculopathy. Correlations with pain and neurologic signs and its value for assessing symptomatic severity Spine (Phila Pa 1976), 1994. 19(21): pp. 2443-9; discussion 2449-50. 7846599

[23] Thomas D (1990). Infrared thermographic imaging, magnetic resonance imaging, CT scan and myelography in low back pain. Br J Rheumatol.

[24] LaBorde TC (1989). Thermography in diagnosis of radiculopathies. Clin J Pain.

[25] Park TY (2020). Hyperthermia associated with spinal radiculopathy as determined by digital infrared thermographic imaging. Med (Baltim).

[26] Cohen JF (2016). STARD 2015 guidelines for reporting diagnostic accuracy studies: explanation and elaboration. BMJ Open.

[27] Konstantinou K, Dunn KM (2008). Sciatica: review of epidemiological studies and prevalence estimates. Spine (Phila Pa 1976).

[28] Mills GH (1986). The evaluation of liquid crystal thermography in the investigation of nerve root compression due to lumbosacral lateral spinal stenosis. Spine (Phila Pa 1976).

[29] Chafetz N, Wexler CE, Kaiser JA (1988). Neuromuscular thermography of the lumbar spine with CT correlation. Spine (Phila Pa 1976).

[30] Pochaczevsky R (1982). Liquid crystal thermography of the spine and extremities. Its value in the diagnosis of spinal root syndromes. J Neurosurg.

[31] Barr K (2013). Electrodiagnosis of lumbar radiculopathy. Phys Med Rehabil Clin N Am.

[32] Datta S (2013). Diagnostic utility of selective nerve root blocks in the diagnosis of lumbosacral radicular pain: systematic review and update of current evidence. Pain Physician.

[33] Byun WM, Ahn SH, Ahn MW (2012). Value of 3D MR lumbosacral radiculography in the diagnosis of symptomatic chemical radiculitis. AJNR Am J Neuroradiol.

[34] Kim JH (2018). Diagnostic accuracy of diagnostic imaging for lumbar disc herniation in adults with low back pain or sciatica is unknown; a systematic review. Chiropr Man Th.

[35] Klineberg E (2015). Diagnosis, treatment, and complications of adult lumbar disk herniation: evidence-based data for the healthcare professional. Instr Course Lect.

